# P-254. The Effect of Disinfection with 222-nm Ultraviolet C on Surfaces Contaminated with *Clostridioides difficile* and Vancomycin-resistant Enterococci in the Bathrooms of Isolation Patients

**DOI:** 10.1093/ofid/ofae631.458

**Published:** 2025-01-29

**Authors:** Hiroki Kitagawa, Kayoko Tadera, Keitaro Omori, Toshihito Nomura, Norifumi Shigemoto, Hiroki Ohge

**Affiliations:** Hiroshima University Hospital, Hiroshima, Hiroshima, Japan; Hiroshima University Hospital, Hiroshima, Hiroshima, Japan; Hiroshima University Hospital, Hiroshima, Hiroshima, Japan; Hiroshima University Hospital, Hiroshima, Hiroshima, Japan; Hiroshima University Hospital, Hiroshima, Hiroshima, Japan; Hiroshima University Hospital, Hiroshima, Hiroshima, Japan

## Abstract

**Background:**

Contaminated environmental surfaces are important sources of transmission for healthcare-associated pathogens, including *Clostridioides difficile* (CD) and vancomycin-resistant enterococci (VRE). Toilet surfaces are easily contaminated by pathogens excreted in stools. In this study, we investigated the efficacy of a 222-nm ultraviolet C (UVC) disinfection device, Care222®, for removing bacterial contamination in the bathrooms of isolated patients.

**Methods:**

This study was conducted in the rooms of five patients diagnosed with *C. difficile* infection and in the rooms of five patients colonized with VRE. The Care222® device is mounted on a mobile cart and fixed at a 45-degree downward angle. The Care222® emission window was placed 1 m from the farthest end of the toilet seat and at a height of 1.1 m from the floor. At this position, UVC irradiation was performed for 45 min at a total 222-nm UVC fluence of 27 mJ/cm^2^. Before and after UVC irradiation, samples were collected from the toilet seat and control panel of the electric warm-water bidet toilet seat, in each room, and colony-forming units (CFUs) were determined.

**Results:**

Disinfection with 222-nm UVC resulted in a significant decrease in the number of CD CFUs compared to the number of CFUs before disinfection (Median CFUs (range), 8 (0–51) vs. 1 (0-5), P=0.004). It also resulted in a significant decrease in the number of VRE CFUs compared to the number of CFUs before disinfection (Median CFUs (range), 7 (1–32) vs. 1 (0-3), P=0.001).

**Conclusion:**

The disinfection with the 222-nm UVC device significantly reduced CD and VRE surface contamination in the bathrooms of isolated patients. Further studies are required to investigate the effectiveness of 222-nm UVC disinfection for reducing environmental pathogen contamination.

**Disclosures:**

**All Authors**: No reported disclosures

